# Neuroprotective function of microglia in the developing brain

**DOI:** 10.1042/NS20200024

**Published:** 2021-01-22

**Authors:** Yuki Fujita, Toshihide Yamashita

**Affiliations:** 1 Department of Molecular Neuroscience, Graduate School of Medicine, Osaka University, 2-2, Yamadaoka, Suita, Osaka 565-0871, Japan; 2 WPI Immunology Frontier Research Center, Osaka University, 3-1, Yamadaoka, Suita, Osaka 565-0871, Japan; 3 Graduate School of Frontier Bioscience, Osaka University, 2-2, Yamadaoka, Suita, Osaka 565-0871, Japan; 4 Department of Neuro-Medical Science, Graduate School of Medicine, Osaka University, 2-2, Yamadaoka, Suita, Osaka 565-0871, Japan

**Keywords:** brain, cell death, microglia, neuron, neurotrophic factors

## Abstract

Microglia are the resident immune cells of the central nervous system and are important for immune processes. Besides their classical roles in pathological conditions, these cells also dynamically interact with neurons and influence their structure and function in physiological conditions. Recent evidence revealed their role in healthy brain homeostasis, including the regulation of neurogenesis, cell survival, and synapse maturation and elimination, especially in the developing brain. In this review, we summarize the current state of knowledge on microglia in brain development, with a focus on their neuroprotective function. We will also discuss how microglial dysfunction may lead to the impairment of brain function, thereby contributing to disease development.

## Introduction

Microglia are resident immune cells in the central nervous system (CNS). They have long been studied for their roles in pathological conditions because of their rapid response and activation with changes in their morphological and functional characteristics, such as their release of inflammatory cytokines and becoming actively phagocytic [1–3]. More recent studies using new genetic and functional approaches have identified roles for microglia in the physiological condition extending far beyond their function as immune cells, especially in the context of CNS development. Advances in *in vivo* imaging studies have demonstrated that microglia can directly communicate with neurons in both healthy and diseased brains. Microglia continuously scan their surrounding environment through extensive processes under physiological conditions [4–8]. The interactions between microglia and neurons contribute to the maintenance of homeostasis of the CNS and are required for the construction of neural circuits during brain development.

During development, neurons construct neural circuits through the seamless progression of a discrete series of events. Once differentiated from progenitor cells, neurons migrate to specific areas, extend their axons toward possible target cells, and establish circuits via the formation of synapses. Initially, neurons form an excessive number of branches and synapses. Inappropriate connections are removed by axon pruning and/or synapse elimination. These formations and regressive processes are both essential for the construction of proper neural circuits. Besides neuron-intrinsic mechanisms, the interactions of neurons with various cell types, such as glial cells and vascular cells, are also involved in these events [9,10]. Recent studies have revealed that microglia play key roles in these processes of establishing neural circuits in the developing brain.

In this review, we will summarize the current knowledge of the role of microglia in CNS development, highlighting the underlying signaling mechanisms, and discuss the significance of the neuroprotective functions of microglia.

## Origin of microglia

Based on Cajal’s silver carbonate staining, del Rio-Hortega had already proposed the concept of a mesodermal origin of microglia [11–13]. Nevertheless, there has been much debate regarding the origin of microglia for many years [14–16]. There have been three main theories in this regard, which hypothesize that microglia originate from (1) mesodermal/mesenchymal tissues [17,18], (2) the neuroectoderm (similar to neurons, astrocytes, and oligodendrocytes) [19,20], or (3) circulating blood monocytes [21]. More recent studies have shown that microglia are derived from myeloid progenitors in the mouse embryonic yolk sac at embryonic day (E)7.5 and enter the brain thereafter at E9.5 [14,22–27].

**Figure 1:**
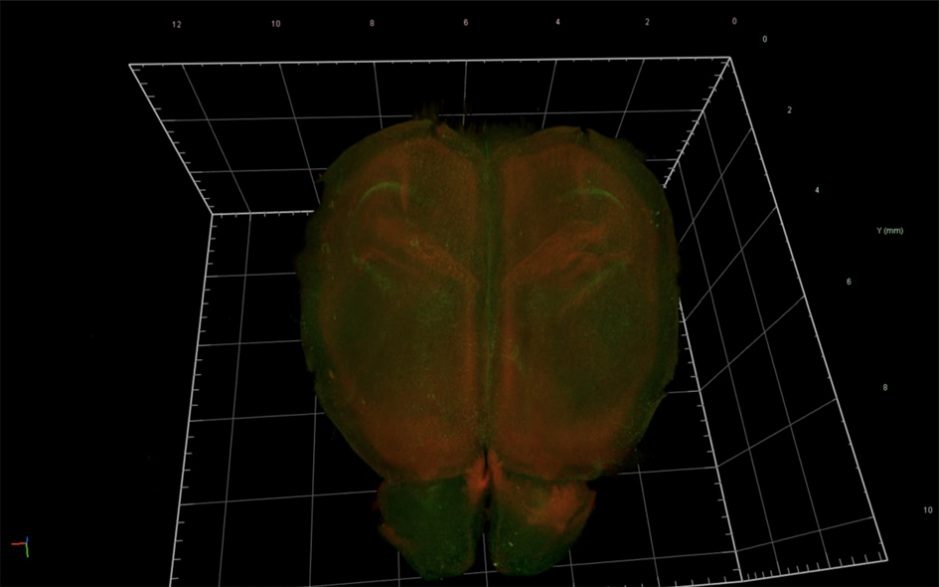
Distribution of microglia in the cerebrum A reconstructed image of the cerebrum cleared by CUBIC L/R+. GFP-expressing microglia (green) and tomato-expressing neurons (red) in the cerebrum from an early postnatal triple-transgenic reporter mouse (CX3CR1^+/GFP^; Rbp4-Cre; Ai14). See Supplementary Material for the video version.

**Figure 2:**
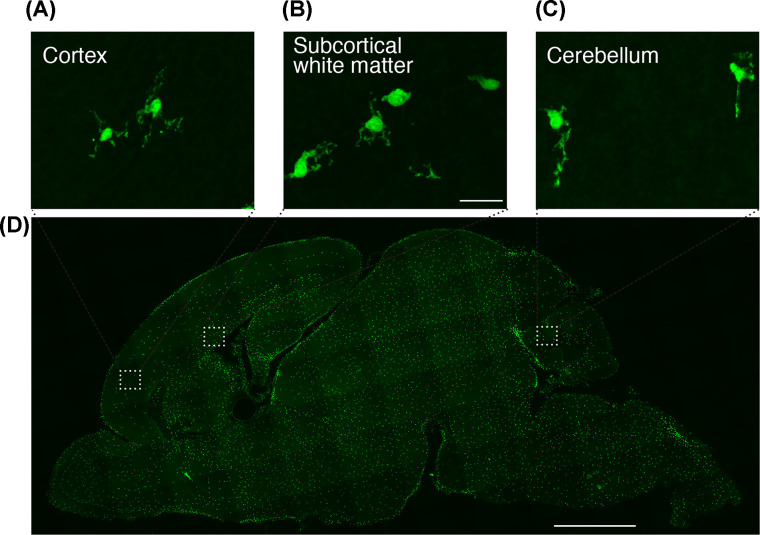
Morphology of microglia in the cortex and axons (**A**–**C**) GFP-expressing cells in the cortex (A), the subcortical white matter (B), and the cerebellum (C). (**D**) Sagittal brain section of a CX3CR1^+/GFP^ mouse at postnatal day 3. Scale bars: 20 μm (A–C), 1 mm (D).

Once microglia reach the brain, they are found throughout the brain (Figure 1, Supplementary Material) but in a specific and heterogeneous colonized distribution. Particularly, microglia accumulate around white matter in the early postnatal brain, forming the ´fountain of microglia’ [28–31]. These microglia show an amoeboid morphology, which differs from their ramified morphology in the adult brain (Figure 2) [28,29,32–34].

## Multiple roles of microglia in brain development

As mentioned above, the typical distributions and activated morphologies of microglia point to specific roles during brain development. Indeed, microglia have been shown to contribute to the regulation of neuronal numbers and the refinement of neural circuits.

## Microglia control proper cell numbers

### Microglia support neurogenesis

Microglia are densely populated in neurogenic niches, such as the subventricular zone (SVZ) in the rodent brain [35,36]. In the mouse embryonic brain, the expression of the chemokine CXCL12 in basal progenitors is involved in microglial recruitment into the ventricular zone (VZ)/SVZ [37]. The overexpression of CXCL12 enhances the recruitment of microglia into the VZ/SVZ; however, it does not affect the proliferation of microglia. Administration of the CXCR4 antagonist, the chemokine receptor for CXCL12, abolishes this response, suggesting that the CXCL12/CXCR4 chemokine-chemokine receptor pair signaling mediates microglia recruitment into the VZ/SVZ.

In addition, a recent study has demonstrated that the CXCL12/CXCR4 system is important for microglial migration in the cortical plate of the developing mouse cortex to assist proper differentiation of post-migratory cortical neurons [38]. The intrapallial microglia distribute homogenously until E14 in mice, but they temporarily disappear from the cortical plate where postmigratory neurons accumulate from E15 to E16. This transient exit of microglia from the cortical plate is regulated by the CXCL12/CXCR4 system. Artificial exposure of cortical plate neurons to excessive microglia causes disturbance of the expression of subtype-associated genes essential for neuronal proper differentiation. These findings indicate that transient distancing of microglia from the cortical plate at the mid-embryonic stage is required for the proper establishment of functional cortical circuit.

It has been reported that microglia support the production of neural precursors in the SVZ, possibly through the release of several different cytokines during normal development [39]. In the rat forebrain, microglia have an active morphology within the SVZ from postnatal day (P)1 to P10, which is then transformed to a ramified morphology after P14. Microglia in the early postnatal SVZ promote neurogenesis, possibly via the release of cytokines, such as interleukin (IL)-1β, IL-6, tumor necrosis factor α (TNFα), and interferon γ. The inhibition of microglia by a microglial inhibitor, such as minocycline treatment from P2 to P5, reduced the number of neuronal progenitors in the SVZ and suppressed the release of IL-1β, IL-6, TNF-α, or interferon γ from activated microglia [39].

Thus, these findings support the idea that microglia can be involved in regulating the production of neuronal cells in the developing brain through the release of key factors.

### Microglia support neuronal survival in the postnatal brain

Microglia also release factors with neurotrophic functions [40–43]. We demonstrated that microglia in the subcortical white matter abundantly express the trophic factor, insulin-like growth factor 1 (IGF1), and support the survival of layer V neurons in the postnatal brain [34].

**Figure 3:**
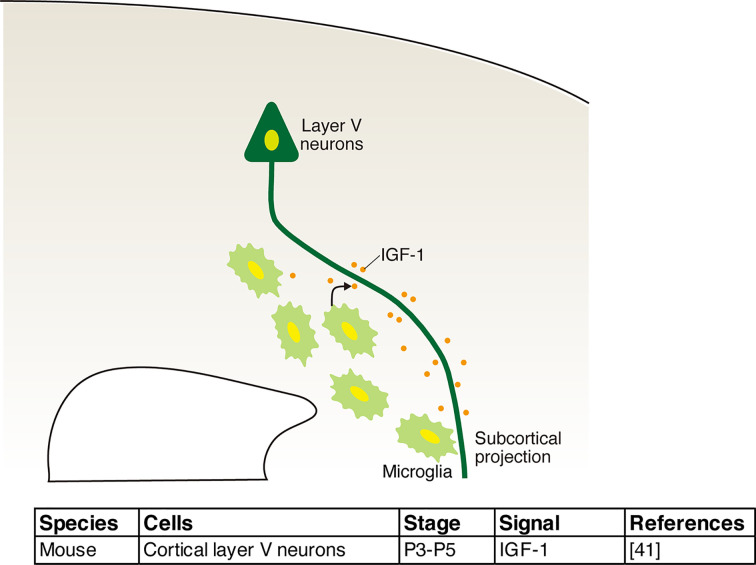
The role of microglia in neuronal survival Microglia that accumulate around the axons from layer V neurons peak at postnatal days 3–7 and support the survival of layer V neurons through the release of insulin-like growth factor 1 (IGF-1).

In the postnatal brain, microglia abundantly accumulate in the white matter, including the subcortical white matter, the internal capsule, and the cerebral peduncle, through which the axons from layer V neurons pass. This accumulation peaked at P3-7, with microglia gaining an amoeboid morphology, and the colonized distribution became increasingly diffuse after P14. The inactivation or depletion of microglia through minocycline treatment in CD11b-DTR transgenic and CX3CR1^−/−^ mice results in increased levels of apoptosis of layer V neurons at P3–5. This developmental stage is consistent with the period characterized by peak microglial accumulation near the axons of layer V neurons. Netrin-G ligand-1 (NGL1) mediates the accumulation of microglia around the axons of layer V neurons expressing its receptor Netrin-G1 [44]. Conditional knockout of netrin-G1 by *in utero* electroporation or in NGL1 ^-/-^ mice results in the accumulation of microglia around the internal capsule through which axons from layer 5 neurons pass. We also identified microglia-derived IGF1 as a survival factor for layer V neurons in postnatal periods [34]. The pharmacological inhibition of IGF1 by H-1356 or the siRNA-mediated knockdown of IGF1 induced apoptosis in layer V, whereas the intraventricular administration of IGF1 rescued minocycline-induced cell death in layer V. In addition, both *in vitro* and *in vivo* analyses have shown that IGF1 also promote axon outgrowth for corticospinal motor neurons [45]. These findings suggest that microglia support neuronal survival and contribute to the establishment of neuronal wiring in the postnatal brain (Figure 3).

Microglia-derived IGF1 also contributes to neuroprotection following CNS injury. The number of IGF1-expressing microglia was increased in the SVZ at 2, 6, and 16 weeks after stroke in the rat middle cerebral artery occlusion model [46]. This long-term accumulation of microglia with a neuroprotective phenotype might be involved in continuous neurogenesis after stroke. Furthermore, microglia cross-talk with other glial cells to exert their neuroprotective functions. Microglia induce astrocyte proliferation via IGF-1 and support neuroprotective scar formation in a mouse spinal cord injury model [47]. Microglial depletion after an injury disrupts glial scar formation, enhances immune infiltration, exacerbates axonal damage, and impairs locomotor recovery [47,48]. Thus, microglia still have a neuroprotective role in pathological conditions.

### Microglia mediate cell death and the removal of damaged cells

In the above sections, we summarized the functional roles of microglia, which increase or influence neuronal numbers. In addition to these progressive events, regressive events, such as cell death, are also important processes in the regulation of appropriate neuronal numbers. Programmed cell death (PCD) is defined as the spatially and temporally reproducible and species-specific loss of large numbers of individual cells during development [49]. PCD may occur both in proliferating and post-mitotic neurons and is a regular feature of normal development rather than an unexpected phenomenon. This regressive event is essential for the removal of excess neurons and regulates the proper number of cells to establish the proper size of the brain alongside proper neural network formation.

One prominently described function of microglia is the removal of damaged cells via engulfment [1].

Microglia are attracted by apoptotic cells expressing ‘find-me’ signals that are exerted at the early stages of apoptosis to recruit phagocytes [50] as well as ‘eat-me’ signals that allow microglia to phagocytose them [51]. In addition, increased rates of cell death trigger microglial proliferation in the mouse VZ/SVZ at E16.5 [37]. Increased neuronal death by the overexpression of the suicide gene thymidine kinase followed by treatment with Ganciclovir or in FoxG1^−/−^ mice resulted in an increased number of proliferating microglia as well as Iba1 and EdU-double positive cells.

These observations suggest that damaged cells recruit microglia, leading to their own removal via microglial phagocytosis. This enables cell death to control the distribution and proliferation of microglia, contributing to further progressive events key to neural circuit formation [52].

In addition to neurons attracting microglia, microglia actively induce neuronal cell death.

**Figure 4:**
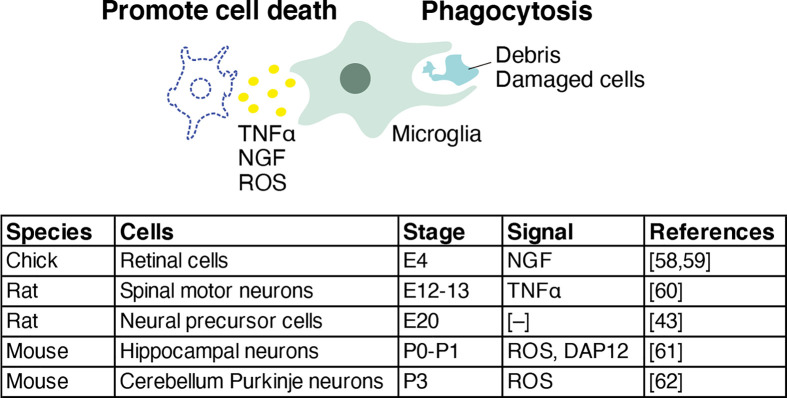
The role of microglia in cell death and the removal of damaged cells Microglia phagocytose damaged cells and induce apoptosis through mechanisms that include the secretion of nerve growth factor (NGF), tumor necrosis factor α (TNF α), and reactive oxygen species (ROS).

Several studies have reported that microglia release factors associated with the promotion of cell death (Figure 4).

In the chick retina, microglia-derived nerve growth factor (NGF), a ligand for p75 neurotrophin receptor, triggers cell death at the early embryonic developmental stages [53,54]. Immunohistochemistry revealed that NGF-immunoreactive cells exist within the neuroepithelium and vitreal surface of the E4 chick retina, and NGF gene expression was confirmed in purified retinal microglial cells. The peptide to prevent p75 neurotrophin receptor-dependent cell death completely blocked NGF-induced cell death in cultured retinal cells.

Other roles of microglia in the cell death of developing neurons are revealed in various animal models. In the rat embryonic spinal cord explants, macrophage-derived TNFα signals differentiating motoneurons expressing TNF receptor 1 to die between E12 and E13, prior to PCD [55]. At the onset of the developmental cell death, amoeboid-shaped microglia accumulate within the lateral motor columns and are in contact with activated-caspase-3 expressing dying motoneurons, suggesting that microglial cells play an active role in motoneuronal PCD [56].

In the mouse hippocampus, microglia-derived superoxide ions mediate neuronal death during neonatal stages from postnatal day (P)1 to P2 [57]. During these stages, microglial processes are in contact with most of the apoptotic cells, and a deficiency in CD11b, which is a subunit of β2-integrin whose expression is restricted to microglia in the brain, or a deficiency in DNAX-activating protein of 12 kDa (DAP12), which is an adaptor containing an immunoreceptor tyrosine-based activation motif whose expression is restricted to developing microglia, decreases neuronal apoptosis and the production of superoxide ions.

In mouse cerebellar slices, microglia also induce Purkinje cell apoptosis by releasing superoxide ions at P3 [58].

This study has demonstrated that the phagocytic activity of microglia can trigger neuronal death [58]. Microglia in cultured cerebellar slices prepared from P3 mice show an amoeboid morphology. The presence of calbindin-positive inclusions inside microglial cells strongly suggested that cerebellar Purkinje cells are engulfed by microglia.

Eliminating microglia by the intraventricular injection of liposomal clodronate into rat embryos at E20 showed an increased number of Pax6-positive neural precursor cells in the SVZ [36]. In the proliferative zones of the developing cerebral cortex in macaques at the prenatal stage (E80), most neuronal precursor cells targeted by microglia in the cortical proliferative zones did not show any signs of cell death or apoptosis, as defined by cleaved caspase-3, TUNEL, phosphatidylserine expression, or nuclear breakdown. This thereby suggested that microglia can phagocytose viable neural progenitors, independent of apoptosis [36,59].

Consistent with these findings, during Drosophila embryogenesis, the phagocytic ability of embryonic glial cells is determined by the expression of phagocytic receptors for apoptotic cells, SIMU and DRPR, and might be established independently and ahead of PCD [60].

Such engulfment-promoted cell death has also been reported in *Caenorhabditis elegans* [61,62].

These observations suggest that PCD is actively promoted by microglial phagocytic ability, and microglia primarily control the number of neural precursors during normal embryonic development.

Collectively, microglia have multiple functions in developmental cell death and survival. They support neurogenesis and neuronal survival by releasing key factors, whereas they remove damaged cells and even induce cell death by releasing pro-apoptotic factors and phagocytizing viable neurons. Although microglial function on PCD seems to be harmful to brain development, both functions of microglia, in the context of producing and removing cells, are indispensable to control the proper number of neurons essential for normal brain development. The presence of a bidirectional cross-talk between neural progenitor cells and microglia in the developing brain may contribute to regulating proper neurogenesis during development.

## Microglia support early wiring

In addition, microglia also support neural circuit assembly. In the embryonic brain, microglia transiently accumulate around specific axonal tracts, including dopaminergic axons and corpus callosum axons, and regulate axon outgrowth and fasciculation. Microglia are also involved in the pruning of excessive axons. Thus, microglia support the establishment and refinement of early wiring.

In the embryonic ventral telencephalon, microglia particularly accumulate around dopaminergic axons [63]. Microglia depletion through Pu.1^−/−^ mutants or CSF-1R blockade by the administration of the anti-CSF-1R antibody to CX3CR1^GFP/+^ mice resulted in an exuberant extension of tyrosine hydroxylase (TH)-positive dopaminergic axons into the subpallium of the embryonic ventral telencephalon. However, no defects in neighboring serotoninergic fibers and internal capsules were observed. In contrast, maternal immune activation, which triggers an immune activation of embryonic microglia, induced a mild but robust reduction in the extension of TH-positive axons into the subpallium at E14.5. In addition, 3D reconstruction of confocal images and electron microscopy revealed a direct contact between microglia and TH-positive axons. TH-positive axon fragments were also detected inside the cytoplasm of GFP-positive microglia, implying that microglia might phagocytose fragments of dopaminergic axons in physiological conditions. These observations suggest that microglia regulate axon outgrowth preferentially to dopaminergic fibers in the embryonic brain via its phagocytic ability.

Microglia are also in contact with developing axonal fibers in the corpus callosum and mediate their fasciculation in the embryonic brain. The loss of DAP12, a key microglial-specific signaling molecule, microglial depletion in Pu.1^−/−^ mice, and a model of maternal immune activation resulted in the defasciculation of dorsal callosal axons at E17.5 [64]. Interestingly, the time-course of microglial accumulation assessed in this study is slightly late compared to the accumulation that of in dopaminergic axons.

Collectively, microglia accumulate in specific axons at different time periods during development. In so doing, they play a key role in early wiring.

## Microglia support the refinement and maturation of neural circuits

Furthermore, microglia have also been reported to contribute to the maturation and refinement of synaptic networks in the postnatal period.

It has been reported that microglia release factors associated with the formation and maturation of synapses [6,8,65,66].

Killer cell activating receptor-associated protein (KARAP)/DAP12 is a transmembrane polypeptide associated with cell-surface receptors, which is detected only in microglia but not in neurons or other glial cells. In addition, microglial KARAP/DAP12-mutant mice show impaired synaptic function [67]. KARAP/DAP12 functional deficiency resulting in long-term potentiation was enhanced and was partly N-methyl-d-aspartic acid receptor-independent in the hippocampal CA1 neurons.

In addition, microglia mediate synaptic pruning to ensure the proper maturation of excitatory synaptic transmission. The frequency and amplitude of miniature excitatory postsynaptic currents are increased in CX3CR1-null mice as compared with those in wild-type littermates in postnatal CA1 hippocampal pyramidal neurons [68]. The long-term depression of excitatory synaptic transmission at CA1 synapses is enhanced in Cx3CR1-deficient mice [69]. Thus, microglia regulate the normal development of glutamatergic synapses in the hippocampus.

Microglia also phagocytose synapses, which has been most elegantly demonstrated in the dorsolateral geniculate nucleus of the thalamus. During the early postnatal period, microglia phagocytose weak retinal ganglion synapses in the dorsolateral geniculate nucleus by recognizing complement component 3, which is abundantly expressed in synapses, through the complement receptor 3 [70–72].

Furthermore, in postnatal layers 2–3 of the somatosensory cortex, microglia induce the formation of new spines [73]. *In vivo* two-photon imaging of mice has demonstrated that the interaction between microglial processes and dendritic shafts of pyramidal neurons prominently mediates the formation of filopodia. This filopodial formation occurs only around P8 to P10, when synaptogenesis occurs abundantly in upper cortical layers.

Moreover, microglial depletion impairs the turnover of spines in pyramidal neurons of the motor cortex, with an effect on both the elimination and formation of spines in both late postnatal (P19) and young adult (P30) mice [74]. Since the depletion of brain derived neurotrophic factor specifically in microglia reproduces the effects on synaptic plasticity, microglia-derived brain derived neurotrophic factor is the key factor involved in the mechanism underlying this effect.

Overall, proper microglia function is required for the normal functional maturation and refinement of neural circuits in the postnatal brain.

## Concluding remarks

In this review, we have highlighted recent observations regarding the origin and colonized distribution of microglia, alongside the multiple roles played by microglia in physiological brain development. In addition to their roles as immune cells in response to inflammation and the clearance of debris by phagocytosis in the pathological condition, microglia play an active role in neuronal circuit formation and maturation in physiological conditions, particularly in developmental stages. They contribute to the control of neuronal survival and PCD, as well as synapse formation and elimination during the maturation of neuronal circuits. *In vivo* imaging studies of awake mice have revealed direct interactions between microglia and synaptic elements in the healthy brain. In addition, some molecular mechanisms underlying these interactions between microglia and neurons have been identified, such as those via the microglial chemokine receptor Cx3CR1. Such indispensable physiological roles of microglia suggest that microglial dysfunction may contribute to neurodevelopmental diseases [75,76]. Indeed, several studies have found that microglial dysfunction has been implicated in the pathogenesis of hereditary disorders, as well as psychiatric symptoms, including depression, anxiety, aggressiveness, and severe dementia [77,78]. These findings suggest that targeting microglial function and the induction of the neuroprotective potential of microglia may represent a good therapeutic approach. However, the mechanisms and physiological functions underlying the interaction between microglia and neurons remain to be elucidated. Recent studies using single cell RNA-seq have shown that transcriptomic features of prenatal microglia and microglia derived from disease animal model are more heterogeneous than those of adult microglia under physiological conditions, reflecting the complexity of microglia functions in the developing and diseased brain [79,80]. Advances in technical approaches, such as the characterization of microglia at a single cell level and the selective gene modification of microglia, will certainly help to more specifically address how microglia affect neural circuit formation and disease pathogenesis.

## Supplementary Material

Supplementary video

## Abbreviations

CNS: central nervous system
DAP12: DNAX-activating protein of 12 kDa
E: embryonic day
IGF1: insulin-like growth factor 1
IL: interleukin
KARAP: killer cell activating receptor-associated protein
NGL1: Netrin-G ligand-1
P: postnatal day
PCD: programmed cell death
SVZ: subventricular zone
TH: tyrosine hydroxylase
TNFα: tumor necrosis factor α
VZ: ventricular zone
